# Probiotics for the Control of Parasites: An Overview

**DOI:** 10.1155/2011/610769

**Published:** 2011-09-28

**Authors:** Marie-Agnès Travers, Isabelle Florent, Linda Kohl, Philippe Grellier

**Affiliations:** Team Adaptation of Protozoa to their Environment, UMR 7245 CNRS, National Museum of Natural History, CP52, 61 rue Buffon, 75231 Paris Cedex 05, France

## Abstract

Probiotics are defined as live organisms, which confer benefits to the host. Their efficiency was demonstrated for the treatment of gastrointestinal disorders, respiratory infections, and allergic symptoms, but their use is mostly limited to bacterial and viral diseases. During the last decade, probiotics as means for the control of parasite infections were reported covering mainly intestinal diseases but also some nongut infections, that are all of human and veterinary importance. In most cases, evidence for a beneficial effect was obtained by studies using animal models. In a few cases, cellular interactions between probiotics and pathogens or relevant host cells were also investigated using in vitro culture systems. However, molecular mechanisms mediating the beneficial effects are as yet poorly understood. These studies indicate that probiotics might indeed provide a strain-specific protection against parasites, probably through multiple mechanisms. But more unravelling studies are needed to justify probiotic utilisation in therapeutics.

## 1. Introduction

**Figure 1:**
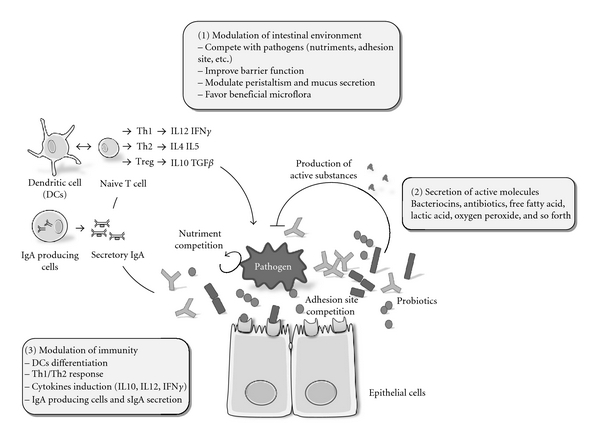
Schematic representation of the different routes by which probiotics may control a pathogen. (1) Probiotics can modulate their physicochemical environment (nutrients, mucus, receptors availability on epithelial cells, pH, tight junctions, and peristaltism). (2) Probiotics can produce biologically active molecules such as bacteriocins, antibiotics, or oxygen peroxide that possess antimicrobial properties. (3) Probiotics can induce immune modulation, either through interaction with dendritic cells that can, in turn, modulate the differentiation of naïve T cells into Th1, Th2, or Treg lymphocytes, leading to different cytokine induction and/or through a humoral immune response *via* IgA producing cells and their secretory IgA (sIgA).

Probiotics have been defined by WHO as “live organisms which when administered in adequate amounts confer a health benefit to the host” (http://www.who.int/foodsafety/publications/fs_management/probiotics2/en/). Importantly, a general probiotic statement on a genus or a species cannot be established, as two closely related strains can induce inverse effects. Therefore, a probiotic must always be fully characterised at a strain level [1]. A good probiotic strain should confer a beneficial property (immune stimulation, protection against pathogens, metabolism, etc), be nonpathogenic, resistant to low pH and acids, thereby persisting in the intestine, and able to adhere to the gut epithelium [2]. About 50 strains corresponding to 26 species fulfil these criteria. Most probiotic organisms are gram positive bacteria, isolated from the human gut microflora or various dairy products such as curd, lassi, and kulfi. However, probiotic beneficial effects have been more often demonstrated in model animals than by direct clinical evidences and depend largely on the dose ingested. Dose of at least five billion colony forming units per day for at least 5 days is recommended [2]. This minimum dose takes into account the survival capacity of the ingested probiotics in the gastrointestinal tract, where they are in competition with the resident bacteria [3]. Three main benefits are reported (Figure 1). 

Modulation of the intestinal environment, by probiotics having the capacity to control the proliferation of surrounding microorganisms and/or by competition for the occupancy of a common biotope (e.g., access to nutriments) [2]. For example, iron is a limiting nutriment: it is essential for most bacteria, and probiotics can compete for its availability. *Lactobacillus* can render iron unavailable for pathogenic microorganisms, either by binding ferric hydroxide on its surface [4] or by secreting siderophores that chelate and transport iron [3]. Some probiotics are also able to influence the composition and equilibrium of the gut microflora (For review, see [5]). For example, probiotic therapy using a mixture of probiotics (VSL#3) was shown to increase the total number of intestinal bacteria and to restore the diversity of the bacterial microbiota in patients [6]. Finally, probiotics can also control their biotic environment through regulation of intestinal motility and mucus secretion [2]. 

Secretion of active molecules (e.g. bacteriocins, antibiotics, free fatty acids, hydrogen peroxide) that can control growth and/or survival of surrounding microorganisms. Bacteriocins are secreted peptides or proteins that generally kill closely related bacteria by permeabilizing their membranes or by interfering with essential enzymes (For review, see [5]). Many of them are produced by *Lactobacillus* probiotic strains (lactacin B, lactacin F, nisin, etc.). *Lactobacillus reuteri* produces reuterin (3-hydroxypropionaldehyde), a broad-spectrum antibiotic, active against bacteria, yeast, fungi, protozoa, and viruses [7]. By lowering the local intestinal pH with lactic acid, probiotics can also modify the growth of acid-sensitive organisms [5]. 

Modulation of immunity, by stimulating the host immune response to a variety of pathogens. In the gut, probiotics interact with the epithelial cells, Peyer's patches M cells, and immune cells. These interactions result in an increase in the number of IgA producing cells accompanied by production of IgM and secretory IgA which are particularly important in mucosal immunity, contributing to the barrier against pathogenic organisms [8, 9]. In addition, probiotics can also affect dendritic cells, which are responsible for collection of antigens from gut and their presentation to naïve T cells, leading to their differentiation to T-helper (Th1, Th2) or T-regulatory lymphocytes. Probiotic molecules implicated in dendritic cell induction are poorly characterized, one exception being the S layer protein A of* L. acidophilus* NCFM that regulates maturation of dendritic cells and T cell functions [10]. Probiotics have also been shown to modulate cytokine release (TNF-*α* IFN-*γ*, IL-10, IL-12) [11]. These cytokines play a central role in maintaining the delicate balance between necessary and excessive defence mechanisms. For instance, polysaccharide A, synthesized by *Bacillus fragilis* NCTC 9343, protects against experimental colitis through an adequate induction of IL-10 production [12]. 

**Figure 2:**
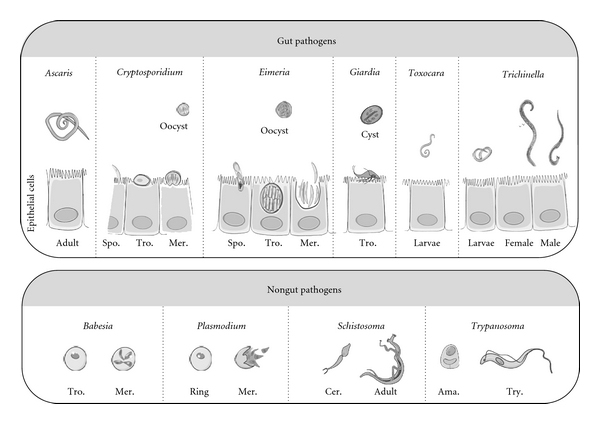
Schematic representation of the different eukaryotic pathogens (gut or nongut pathogens) for which probiotics have been tested. Not to scale. Approximate sizes: adult *Ascaris* up to 30 cm; *Cryptosporidium* oocyst 4 *μ*m; *Eimeria *oocyst 10 *μ*m; *Giardia* cyst 15 *μ*m; adult *Toxocara* up to 20 cm; adult *Trichinella* 3 mm. *Babesia* 5 *μ*m; *Plasmodium* 7 *μ*m, adult *Schistosoma* 16 mm, and *Trypanosoma* trypomastigote 12 *μ*m. Spo.: Sporozoite; Tro.: Trophozoite; Mer.: Merozoite; Cer.: Cercae; Ama.: Amastigote; Try.: Trypomastigote.

In conclusion, probiotics can kill or inhibit pathogens by strain-specific mechanisms relying on competition, molecule secretion, and/or immune induction. Most of the described interactions imply a prokaryotic pathogen that colonizes the same gut compartment. Recently, several studies have investigated whether probiotics could control the proliferation of eukaryotic pathogens, either in the gut as the probiotic, or in a different compartment (Figure 2, Table 1). This concept is supported by recent studies showing that gut commensal microflora can play a critical role in the completion of the life cycle of the intestinal parasitic nematode *Trichuris muris* and in the modulation of the host immune response [13] and can also provide indirect protective immunostimulation against the nongut parasite, *Toxoplasma gondii *[14]. In the following chapters, we will report the recent findings concerning the effects of probiotics on several parasites as illustrated in Table 1 and propose future directions to help to standardize probiotic tests on eukaryotic pathogens. 

## 2. Cryptosporidium

*Cryptosporidium* is an intestinal pathogen belonging to the Alveolata group that can cause devastating gastrointestinal infection in immunosuppressed humans. In the environment, *Cryptosporidium *is found as oocyst, the infective form, in water. After ingestion, the oocysts travel through the gut lumen to the small intestine, where they release the motile sporozoites that adhere and invade the epithelial gastrointestinal cells. The sporozoites focally disrupt the microvilli and penetrate the host cells to establish their intracellular niche, where they remain in an extracytoplasmic vacuole. After parasite replication and evasion, oocysts are generated and excreted in the faeces (for a review, see [15]) (Figure 2). Intestinal epithelial cells, infected by *Cryptosporidium parvum*, show impaired Na^+^ and H_2_O absorption as well as enhanced Cl^−^ secretion, leading to diarrhea. Abnormalities in the barrier properties of the intestinal epithelium, caused in part by the disruption of intercellular junctional complexes, contribute also to diarrhea. Despite a real risk of waterborne outbreaks of cryptosporidiosis, there is no completely efficient therapy available. The most commonly used drugs, such as paromomycin and azithromycin or nitazoxanide, are only effective in combination with immune restoring agents [16]. Immunocompetent adult mice are capable of controling *Cryptosporidium parvum* infections, while IFN-*γ* knockout and severe combined immuno deficiency mice (SCID) are susceptible to this parasite [17, 18]. Besides immunity, the intestinal flora can also influence resistance to *Cryptosporidium *[19]: germ-free adult immunocompetent mice have an increased susceptibility to *Cryptosporidium* whereas SCID mice, colonized with a defined anaerobic flora (Altered Schaedler Flora), are able to resist infection [20]. 

Beneficial effects of probiotics upon cryptosporidiosis have been demonstrated: adult mice (female C57BL/6 immunosuppressed by the murine leukemia virus (strain LP-BM5), 3-4 weeks old) fed daily with *L. reuteri *strains 4000 and 4020 or *L. acidophilus* NCFM presented reduced oocyst shedding [21, 22]. This partial protection was not associated with an immune restoration (cytokines production). Daily ingestion of *L. reuteri* was also efficient to prevent *C. parvum* intestine colonization and tissue lesions in a host with a deficient immune system (gnotobiotic TCR-alpha deficient mice). Waters et al. suggested that protection was due to secretion of as yet unidentified antimicrobial products [23]. In human, a single case of resolution of prolonged cryptosporidiosis by a probiotic treatment was documented [24]. 

On the other hand, probiotics seem less efficient in neonatal animals [25]. No significant benefit could be observed using two different mixtures of probiotics, Actimel or VSL#3 probiotic mixture, although there was a tendency to a shorter duration of infection in probiotic-fed animals. Nevertheless, the authors concluded that one could not rule out some effect in other experimental conditions, such as a prolonged administration before infection and the use of alternative animal models with a mature gut flora and immune system. An absence of effect was also observed under field conditions in Holstein calves, born on a dairy farm where cryptosporidiosis was endemic. In spite of a daily ingestion of a bacterial mixture for 10 days after birth, the incidence of diarrhea and faecal shedding was similar in treated and nontreated animals [26]. One of the limitations of studies in farm animals is the simultaneous exposure to both probiotic and parasite from birth onwards. 

Interestingly, in vitro studies demonstrated the inhibitory effects of cell-free supernatants of *L. acidophilus* NCFM and *L. reuteri* strain 23272 on *C. parvum* and *C. hominis* viability and infectivity [27, 28]. Similarly, cell-free supernatants of *Bacillus brevis*, *Enterococcus faecium*, and *Pseudomonas alcaligenes* reduce *C. parvum* oocyst persistence by inducing oocyst premature excystation [29]. The compounds at the basis of such an inhibition are under investigation. 

## 3. Giardia

*Giardia lamblia* (also known as *Giardia intestinalis* or *Giardia duodenalis*) is an intestinal pathogenic protozoan parasite belonging to the Diplomonad group, that causes ~280 million symptomatic human infections per year [30]. This monoxenous waterborne parasite has the capacity to infect a wide range of hosts. In humans, as little as ten environmentally resistant cysts are sufficient to initiate an infection. The cysts liberate the motile and replicative forms known as trophozoïtes during their gastrointestinal transit. These forms proliferate in the gut lumen, where they adhere to the epithelium (Figure 2). This phase is associated with the symptoms of the disease: watery diarrhea, epigastric pain, nausea, vomiting, and weight loss usually appear 6–15 days after cyst ingestion, but half of the infections remain asymptomatic. Treatments are based on metronidazole and nitroimidazole, but infections may also resolve spontaneously. T cells, neutrophiles, macrophages as well as IgM, IgG, and IgA antibodies are major players of the immune response necessary for resolution of giardiasis. T-cell cytokines may also induce production and release of antigiardial defensins [31]. Some factors, such as common variable immunodeficiency (hypogammaglobulinemy) or altered gut microflora, appear to predispose to *Giardia* infection [29]. Although the clinical impact has been reported to be stronger in young children and undernourished or immunodeficient individuals, there is little insight into how *Giardia* spp. cause the disease since the trophozoites are not invasive and secrete unknown toxins [30]. 

The first proposal of the use of probiotics to control infections by *Giardia* came from the discovery that isogenic mice presented a variable susceptibility to *Giardia* infection depending on their intestinal flora [32]. Interestingly, resistance to *Giardia* infection could be transmitted from mouse to mouse by common housing and was abrogated by using antibiotics, such as neomycin, active against the resident anaerobic flora. 

An important step towards the comprehension of the probiotic activity was the discovery that the culture supernatant of the probiotic strain LA1 of *Lactobacillus johnsonii* was capable of controlling *G. lamblia* growth in vitro [33]. In this study, six other *Lactobacillus acidophilus* strains were also tested but did not show any noticeable effects. *L. johnsonii* LA1 supernatant blocked *G. lamblia* development in G1 phase but was apparently unable to prevent the adhesion of *G. lamblia* to epithelial cells. Biochemical characterization of supernatants revealed the presence of unidentified heat-sensitive low molecular weight product(s) [33]. Daily addition of live bacteria to the drinking water of gerbils, seven days before oral infection by *G. lamblia* cysts, protected the animals [34]. A decrease in the production of cysts in the faeces, a reduction of the prepatent phase and global better health were also observed. This treatment showed protection against the diminished nutrient absorption as well as the histological changes of the intestinal mucosa normally associated with giardiasis. Increased splenocyte responses to T-cell and B-cell mitogens, as well as to *Giardia* antigens, suggested that, in addition to a potential effect of extracellular factors, *L. johnsonii* LA1 treatment reinforced the host immune response against *Giardia*. 

*Lactobacillus casei* MTCC 1423 strain as well as *Enterococcus faecium* SF68 were also effective in eliminating *Giardia* infection from mice [35, 36]. Protection was associated with a diminution of atrophied villi and infiltrating cells in the small intestine of probiotic-treated mice [35] or with an enhancement of the immune response since a production of specific anti-*Giardia* intestinal IgA and IgG was noticed in treated mice [36]. Recently, in vivo experimentation on malnourished mice showed that daily pretreatment with *L. casei* MTCC1423 efficiently reduced both the severity and the duration of giardiasis, compared to nonprobiotic-fed malnourished mice [37]. 

Studies performed in human patients in 1995 indicated the beneficial effect of the administration of the probiotic yeast *Saccharomyces boulardii *(Reflos), not directly to prevent giardiasis but to help in the recovery from postinfection irritable bowel syndrome (PI-IBS, see Section 7), a complication appearing in some patients cured from the parasite by drug treatments [38]. This observation was supported by a later study showing that *S. boulardii* reduced the number of parasite cysts in faeces from patients treated by the combination of *S. boulardii* and the drug metronidazole versus patients treated by metronidazole only [39]. 

While these studies converge towards the existence of a beneficial effect provided by different types of probiotics in giardiasis, additional work needs to be realized to determine whether there is a direct effect on *G. lamblia* development in the host or just a reduction of the pathological effects, or, more likely, a combination of both. 

## 4. Eimeria

*Eimeria* is an apicomplexan parasite responsible for coccidiosis in poultry, livestock, and small animals such as rabbits, dogs, and cats. Avian coccidiosis is a major parasitic disease in poultry, with a very high economical impact [40]. Birds become infected through ingestion of sporulated oocysts that subsequently excyst to form sporozoites in the lumen of the upper intestine. These sporozoites migrate to their preferred sites of development, different between the seven species of veterinary importance. They then invade villi enterocytes and undergo a first asexual multiplication, the schizogony, leading to the release of numerous merozoites that initiate a second schizogony by infecting new epithelial cells. Macro- and microgametes are finally produced, initiating the sexual phase that yields environmental resistant oocysts that are shed in the faeces [41] (Figure 2). Drugs (e.g., amprolium, halofuginone, or polyester ionophores such as monensin lasalocid) and live vaccines are the two main control measures to fight this disease. However, drug resistance has to be managed constantly, and no new drugs have been introduced for many years. Live vaccines against coccidiosis are highly effective, based on nonattenuated and attenuated strains. They show however a low margin of safety for the former and a risk of reversion to virulence as well as expensive large-scale production for the latter. No successful approaches to develop recombinant vaccines have been reported [42]. Alternative control methods are therefore needed [43]. Probiotics have been widely used in the poultry industry, because they help maintaining the normal intestinal microflora, improve feed intake and digestion, and are capable of stimulating the immune system [44]. Only a limited number of studies have addressed their protective effect against the *Eimeria* species responsible for avian coccidiosis [45–49]. 

The administration of Primalac, a commercially available *Lactobacillus*-based preparation, to chicken from birth to three weeks is able to stimulate intestinal intraepithelial lymphocytes and significantly decrease (up to 75% reduction) the number of *E. acervulina* oocysts in the treated chickens [45]. Moreover, in birds fed on a vitamin A deficient diet, Primalac was shown to enhance birds immunity, even if, in this case, oocyst shedding was less reduced than in the previous study (up to 26%) [46]. A third series of experiments with the same general protocol but with a doubled infective dose showed a small but significant elevation of cytokine levels (*γ*-IFN and IL-2) together with a reduction in oocyst shedding in Primalac-treated versus control chicken, but no differences in the anti-*Eimeria* antibody level [47]. It was concluded that the protection level may depend upon the challenge dose but is mediated by immune stimulation as attested by elevation of the cytokines levels [47]. 

Other commercially available probiotic preparations (Mitomax, a combination of *Pediococcus acidilactici* and *Saccharomyces boulardii*, and Mitogrow, *Pediococcus acidilactici *only) were tested in chickens subsequently infected with *E. tenella* and *E. acervulina* using the experimental procedures described by Dalloul et al. [40, 48]. While Mitomax caused a 10–38% reduction in the number of shed oocysts, accompanied with an elevated level of anti-*Eimeria* antibodies [48], Mitogrow caused an elevation of anti-*Eimeria* antibodies and no significant reduction in the number of *E. acervulina *shed oocysts [40]. 

Thus, although the results converge towards the existence of a protective, though partial effect, by these different probiotic preparations against avian coccidiosis, the mechanisms involved remain currently elusive, with variable data concerning the reduction in oocyst shedding, the levels of cytokines, and anti-*Eimeria* antibodies. 

Tierney et al. investigated the interactions between chicken-derived *Lactobacillus* strains and *Eimeria *[49]. Three *Lactobacillus* strains were isolated from different parts of chicken gastrointestinal tract and were tested for their capacity to prevent *E. tenella* invasion in vitro in a MDBK (Madin-Darby bovine kidney) cell model. All strains inhibited the invasion significantly, possibly through steric interference or competitive exclusion. The potential effect of their secreted extracellular factors was further investigated by testing *Lactobacillus* culture supernatants. One supernatant produced by the caecum-derived isolate *Lactobacillus salivarius *Lb16c6 displayed a significant activity that was marginally affected by a 30 min treatment at 100°C. The molecular principle responsible for this inhibitory effect is, however, not yet discovered. 

## 5. Worms

In the course of their work showing that immunostimulants can induce nonspecific resistance against parasites, Bautista-Garfias et al. evaluated the capacity of viable or dead probiotic *Lactobacillus casei* to induce resistance against *Trichinella spiralis* in mice [50]. This nematode is responsible for trichinellosis, one of the most widespread and clinically important diseases in the world. Humans can be infected by eating infected food. Worms mature in the intestine of an intermediate host, such as pig, enter the blood and the lymphatic system and encyst in striated muscles. The migration of larvae causes host tissue damages and inflammatory reactions with complications, which may lead to death. The efficiency of treatments based on mebendazole or albendazole is variable. Both viable and dead *L. casei* ATCC7469 were administrated orally to NIH mice and induced a protective response with a significant reduction of both adult worms (58 and 44%, resp.) and larvae per gram of muscle (up to 70%). Treatment with culture supernatant of *L. casei* was less efficient but still showed a significant effect (32% reduction of adult worms). The authors attribute the protective effect to the production of IL-2, *γ*-IFN, and nitric oxide [50, 51]. 

*Toxocara canis* is an intestinal ascarid that infects primarily dogs. Humans and rodents are paratenic hosts that become infected by ingesting eggs, either on contaminated food or by geophagy. Ingested eggs hatch and spread out throughout the body, leading to symptoms associated with human toxocariasis (visceral or occular larva migrans or covert toxocariasis). Basualdo et al. reported the spectacular effect of *E. faecalis* CECT 7121 on *T. canis* larvae development in N:NIH-Swiss mice: a 90% reduction of the number of larvae in liver and lungs was measured 48 h after infection with embryonated eggs [52]. 

Similarly, *Ascaris suum*, which normally infects pigs, can also be transmitted to humans, where the migrating larvae produce liver lesions and eosinophilic pneumonitis. Probiotic treatment of sows during pregnancy and of their piglets after birth with *Bifidobacterium lactis* (a pig isolate) attenuate the inhibition of glucose absorption in the small intestine induced by *A. suum* infection, a sign associated with the parasite expulsion from the jejunum [53]. Probiotic-treated pigs still expulse *A. suum* normally from the intestine. Treatment appears thus to counteract a negative aspect of the response to infection related to nutrient uptake without affecting the protective immune response [53, 54]. 

*Schistosoma mansoni*, a blood-dwelling trematode worm, is the primary causative agent of bilharziosis. Human infection is initiated during water exposure to the free-swimming fork-tailed cercariae. After maturation in skin, larvae migrate through the skin, blood, lungs, and liver and finally reach the mesenteric venous plexus. Some of the eggs deposited by the female adults pass through the venule walls, cross the intestinal mucosa, and are evacuated with the fecal material. Eggs then infect their intermediate snail host, *Biomphalaria glabrata*. *Zymomonas mobilis*, a bacterium mainly known for its bioethanol-producing capabilities and originally isolated from alcoholic beverages, was reported to provide over 60% protection from the infection of *S. mansoni*, in mice, when orally administrated as a curative treatment (7 days after infection with cercaria) [55]. The degree of protection was determined 60 days after infection, by the number of worms recovered from the murine liver by perfusion. As far as histopathology was concerned, lesions (granulomas) in the liver and the intestine were numerous and similar in the treated and nontreated groups. Eggs were also abundant in the intestine, particularly in the jejunum-ileum part. On the contrary, the administration of *Z. mobilis* as a prophylactic way (7 days before infestation) did not significantly protect from infection, and worse, the combination of prophylactic and curative treatments exacerbated the symptoms. 

## 6. Other Parasites

For other eukaryotic pathogens, the effects of probiotics have mainly been reported by one research group that demonstrated the potential effect of *L. casei* ATCC 7469 in the protection of nongut parasites, such as *Babesia*, *Plasmodium*, or *Trypanosoma* (Bautista and coworkers in Mexico). 

Oral or intraperitoneal treatments of *L. casei* ATCC 7469 in *Babesia microti* (Gray strain) infected mice significantly reduced parasitemia, potentially through a stimulation of the innate immune system [56]. The protective response was improved when the lactobacilli were administered 3 days before or on the same day of parasite infection versus 7 days before. Since in *B. bovis* infections, the early innate response has been attributed to early appearance of IL-12 and *γ*-IFN transcripts in the spleen [57], it has been suggested that *L. casei* could enhance this protective response. The molecular mechanism is currently under investigation, through the testing of low and high molecular components isolated from lactobacilli for their capacity to induce early protective immune response against *B. microti* [58]. 

*L. casei* ATCC 7469 also conferred a protective effect against the malaria parasite *Plasmodium chabaudi* AS in NIH mice [59]. *L. casei *enhanced a nonspecific resistance to *P. chabaudi*, with longer prepatent periods (5 days versus 4 days in control mice), shorter patent periods (8 days versus 11 days in control mice), accompanied by a reduction in parasitemia and viability of parasites recovered from the spleen of treated mice. Nitric oxide concentration was increased (500% to 900%) in serum of *L. casei*-treated mice and was proposed to confer a protective effect upon the plasmodial infection. 

The same probiotic strain also showed a protective effect against *Trypanosoma cruzi* (Ninoa strain), the agent of Chagas disease in NIH mice [60]. Oral or intraperitoneal doses of lactobacilli 7 days before parasite infection showed a significantly reduced parasitemia over the next 50 days. Whether the host immune response, known to be stimulated by *L. casei* [61] and capable of controling *T. cruzi* infections [62], was responsible for this protective effect is still discussed. 

Finally, a beneficial effect of administration of the probiotic yeast *Saccharomyces boulardii* (Ultra-levure) in association with antibiotics was reported in acute amoebiasis due to *Entamoeba histolytica*, with significant decrease of the duration of symptoms (diarrhea, fever, abdominal pain) and presence of cysts in stools [63]. 

## 7. Postinfective Irritable Bowel Syndrome

Irritable bowel syndrome (IBS) is a functional gastrointestinal disorder in which abdominal pain is associated with defecation or alterations in bowel habits in the absence of an organic cause [64]. The pathophysiology of IBS remains elusive but it is generally accepted that symptoms originate from a gut dysfunction and include altered motility in response to stimuli and sensory perception [65, 66]. Low-grade inflammation and immune activation are one proposed mechanism of IBS pathogenesis. The strongest recognized environmental risk factor for IBS development is bacterial gastroenteritis. Postinfective IBS (PI-IBS) patients display similar dysfunctions to those of IBS patients. As gastroenteritis disrupts intestinal microbiota, it has been proposed that correction of gut dysfunctions by commensal microbiota could offer therapeutic potential in PI-IBS patients [65, 67, 68]. Intestinal parasites, such as *Blastocystis hominis*, *Cryptosporidium parvum*, *Giardia lamblia*, *Entamoeba histolytica*, or the nematode *Trichinella spiralis* can cause IBS-like symptoms and may also play a possible role in the IBS etiology [66, 69, 70]. The potential of probiotics to treat IBS-like symptoms associated with parasite infection has been little evaluated. Reliable studies come from the *T. spiralis* model in NIH Swiss mice, which shows similarities to PI-IBS [65]. Adult worms and larvae induce functional alterations (inflammatory response, increased bowel motility, growth of smooth muscle, and modification of mucus production), which persist at least 3 weeks after parasite expulsion. None of the tested probiotics administrated orally (*Lactobacillus paracasei *NCC2461, *L. johnsonii *NCC533 (LA1), *Bifidobacterium longum *NCC2705, and *B. lactis *NCC362) interfered with parasite eviction. However, a strain specific effect was measured on the persistent muscle hypercontractibility observed in this model after recovery from infection: only *L. paracasei* attenuates postinfective hypercontractibility. The beneficial effect is correlated with a modulation of the immunologic response to the parasite and/or a direct or indirect effect of *L. paracasei* on muscle hypercontractibility. The protective activity is also present in *L. paracasei*-culture supernatant and is heat labile, indicating that it is probably a secreted metabolite. *L. paracasei* effects were further investigated using an elegant NMR-based metabolomic approach [71]. *T. spiralis*-infected mice showed altered metabolic profiles, which were related to intestinal hypercontractibility, muscular hypertrophy, and disrupted jejunal functions. *L. paracasei* treatment normalized the muscular activity and the disturbed energy metabolism. The authors concluded that *L. paracasei* treatment may be beneficial in patients with PI-IBS. 

In a gnotobiotic T-Cell receptor-deficient mice model, *C. parvum* induces a persistent infection as well as IBS-like lesions in the caecum. When these mice were pre-colonized by *L. reuteri* and then challenged with *C. parvum*, fewer parasites were detected, and associated hyperplasic and inflammatory lesions were diminished [23, 70]. 

Although clinical evidences of efficacy begin to emerge, the overall impact of probiotics for PI-IBS treatments stays highly debated. This is mainly due to the difficulty to compare the studies because of differences in experiment design, probiotic dose and strains, and responsible agent [67, 68, 72–74]. 

## 8. Probiotic Treatments, an Emerging Therapeutic Strategy in Parasitic Diseases?

The above studies converge towards a beneficial effect of probiotics to control parasitic infections and point towards a strain-specific probiotic effect. As yet, little is known on the cellular or molecular mechanisms sustaining these effects. So far, the experimental studies on *Cryptosporidium*, *Giardia*, or *Eimeria *are the only ones that support a probiotic action *via* the secretion of an active principle that can inhibit parasite development, although the molecular nature of these components remains unknown. Probiotics have been also proposed to influence gut microflora and development of immune response. The underlying mechanisms are however not clear, involving stimulation of different subsets of immune system cells to produce cytokines, which in turn play a role in the induction and regulation of the immune response, and to enhance intestinal IgA immune responses and increase intestinal mucin production (for a review, see [75]). *L. johnsonii *LA1, *Lactobacillus *strain GG, or *Bifidobacterium lactis* Bb12 have all been shown to modulate IgA immune responses [75], whereas *L. rhamnosus *GG increased intestinal mucin production [76]. These actions may boost intestinal clearance of parasites such as *Giardia* and could explain the in vivo protection conferred by some probiotics [31]. Similarly, IL-10 and secretory IgA response, important actors in an efficient anti-*Cryptosporidium* immune response, have been shown to be induced by some probiotic strains [77]. Even if a direct link between probiotic administration, immune effectors induction, and parasite elimination has not been yet clearly established, it seems highly probable. 

Proposing probiotics as alternatives to classical treatments, such as drugs or vaccines, against parasites appears unreasonable; a complementary therapeutic approach to reduce risks of infestation or to sustain classical treatments seems more realistic. For the moment, studies of probiotic effects on parasites are still in their infancy, and further investigations are needed to move forward in this direction. Several important points also need to be addressed.

The probiotics used have to be precisely characterized at a strain level. 

Efforts need to be made to standardize protocols in each model (administration, dose, time, etc.). Absence of probiotic effect on cryptosporidiosis was indeed observed for short probiotic pretreatments (2 days) or concomitant contact with probiotic and pathogen [25, 26] in contrast to longer pretreatments (13 days [21, 22]). But long-term immune stimulation does not increase the effect. In a healthy subject, a great activation of the immune system cannot be conveniently obtained, because constant antigen stimulation could produce negative effects on the host, including autoimmunity [9]. 

More studies need to be realised, combining a larger number of pathogens as well as their corresponding host/animal models, with a greater variety of probiotics (either individually or in combination). 

Another factor of variability that has to be taken into account is the gut microflora of the experimental animal models. While it is obvious that the animal genetic background is important, the environmental factors, such as hygiene conditions, feed quality, and stress, can also affect the established microflora, influencing the results of the studies [78]. To overcome such intrinsic variations, models with identical genetic background (inbred strain) and a controlled microflora, which are subject to the same feeding, are needed. Simplified models such as gnotobiotic animals (mice with a defined microflora) are invaluable tools for exploring effects of gut microorganisms as probiotics on the host [5]. It would also be interesting to monitor the establishment of the probiotics in the gut and its influence on the established microflora. 

In depth understanding of the molecular mechanisms sustaining probiotic action is required to properly design future probiotic treatments. As probiotics can kill pathogens through secretion, inhibit their adhesion or invasion, inactivate toxins, or compete for nutriments, most studies focused on intestinal pathogens with the hypothesis of a local probiotic efficiency. But in vivo studies on nongut pathogens (*Plasmodium*,* Trypanosoma*,* Babesia*, etc.) support a remote effect provided by probiotics probably through a nonspecific immune stimulation. In all cases, a lot of effort needs to go in the elucidation of the mode of action of the promising organisms. 

## 9. Conclusion

The concept that probiotics could control the development of eukaryotic pathogens is emerging. Therapeutic approaches with probiotics could help to reduce the risks of infestation by specific parasites or complement classical anti-parasite treatments. A better understanding of molecular mechanisms underlying the beneficial effects of probiotic on the parasite infection is essential to validate the approach. Further deeper investigations are thus needed using more defined protocols (specific probiotics and experimental models), as well as extended clinical investigations. Gnotobiotics, whose genotype and microbial status can be clearly defined and whose diet and environmental conditions can be easily controlled, could be invaluable tools to go forward in this direction.

## Figures and Tables

**Table 1:** List of probiotics tested on different eukaryotic pathogens. The first column illustrates the pathogens studied; the second one indicates the probiotics tested (and if known, their strain reference; n.s. non specified) and the corresponding references. The third column gives details of the level of the studies: clinical in patients (Human), in vivo in natural or experimental animal models (pig, mouse, calf, rat, chicken, gerbil), or at a cellular level (cyst differentiation and survival, trophozoites development, and invasion capacity). The fourth column specifies the administration conditions of the probiotic (A): a–d: before infection; a—0–3 days, b—3–7 days, c—7–15 days, d—more than 15 days, e—after infection, and f—concomitant administration and infection. The last column indicates the result (R) of the studies. Results are indicated in terms of reduction of parasitic load in comparison to control for in vivo studies or in term of reduction of viability/infectivity for in vitro assays, −: negative impact of probiotic, 0: no significant effect, +: 25–50% of reduction, ++: 50–75% of reduction, and +++: 75–100% of reduction. n.d.: not determined; ∗: clinical case.

Pathogen	Probiotic tested	Host	A	R
* Ascaris suum *	*B. lactis *(pig isolate) [53]	Pig	d	n.d.

*Babesia microti*	*L. casei *ATCC7469 [56, 58]	Mouse	a	+++
			b	+

*Cryptosporidium parvum *	*L. reuteri *4000, 4020 [21]	Mouse	c	+++
	*L. reuteri *4000, 4020 or *L. acidophilus *NCFM [22]	Mouse	c	++
	*L. reuteri *4000, 4020 [23]	Mouse	c	++
	*L. rhamnosus *GG + *L. casei *shirota [24]	Human	e	∗
	VSL#3 or Actimel [25]	Neonatal rat	a	0
	*B. brevis, E. faecium, P. alcaligenes *[26]	Calf	f	0
	*L. reuteri *ATCC23272 or *L. acidophilus *NCFM [27, 28]	Cell culture		+
	*B. breve* ATCC15698 or *B. longum *ATCC15707 [28]	Cell culture		+++
	*B. brevis*, *E. faecium* and *P. alcaligenes* [29]	Cell culture		+++

*Eimeria* *tenella/acervulina *	Primalac [45–47]	Chicken	d	++
	Mitomax [48]	Chicken	d	+
	Mitogrow [40]	Chicken	d	+
	*L. acidophilus *Lb33ac, *L. salivarius* Lb14c7 Lb16c6 [49]	Cell culture		++

*Giardia lamblia*	*L. johnsonii *LA1 [33]	Cell culture		+++
	*L. johnsonii *LA1 [34]	Gerbil	b	++
	*L. casei *MTCC1423 [35]	Mouse	b	+++
	*E. faecium *SF68 [36, 37]	Mouse	b	+++

*Plasmodium chabaudi*	*L. casei *ATCC7469 [59]	Mouse	c	+

*Schistosoma mansoni*	*Z. mobilis *[55]	Mouse	b	++
			e	−

*Toxocara canis*	*E. faecalis *CECT7121 [52]	Mouse	b	+++

*Trichinella spiralis*	*L. casei *ATCC7469 [50, 51]	Mouse	b	+

*Trypanosoma cruzi*	*L. casei *ATCC7469 [60]	Mouse	b	+++

*B. breve: Bifidobacterium breve; B. brevis*: *Bacillus brevis; B. lactis*:* Bifidobacterium lactis; E. faecium*:* Enterococcus faecium; L. acidophilus*: *Lactobacillus acidophilus; L. casei *:* Lactobacillus casei; L. johnsonii: Lactobacillus johnsonii; L. paracasei: Lactobacillus paracasei; L. reuteri*:* Lactobacillus reuteri; L. rhamnosus*:* Lactobacillus rhamnosus; P. alcaligenes*:* Pseudomonas alcaligenes; S. boulardii: Saccharomyces boulardii*;* Z. mobilis: Zymomonas mobilis.*

Sources of commercial probiotics: *Actimel *: *Lactobacillus bulgaricus, Streptococcus thermophilius, and Lactobacillus casei* DN-114 01;* Mitogrow *Imagilin Technology, consists of live *Pediococcus acidilactici; Mitomax* Imagilin Technology, consists of live *Pediococcus acidilactici* and *Saccharomyces boulardii; Primalac *contains primarily *Lactobacillus acidophilus *and *Lactobacillus casei;* VSL#3: four strains of lactobacilli (*Lactobacillus plantarum*, *Lactobacillus casei*, *Lactobacillus acidophilus*, and *Lactobacillus bulgaricus*), three strains of bifidobacteria (*Bifidobacterium infantis*, *Bifidobacterium longum*, and *Bifidobacterium breve*), and one strain of streptococcus (*Streptococcus thermophilius*).

## Abbreviations

IBS: Irritable bowel syndrome
PI-IBS: Postinfective IBS.

## References

[1] Azas-Braesco V, Bresson JL, Guarner F, Corthier G (2010). Not all lactic acid bacteria are probiotics, but some are. *British Journal of Nutrition*.

[2] Gupta V, Garg R (2009). Probiotics. *Indian Journal of Medical Microbiology*.

[3] Oelschlaeger TA (2010). Mechanisms of probiotic actions—a review. *International Journal of Medical Microbiology*.

[4] Elli M, Zink R, Rytz A, Reniero R, Morelli L (2000). Iron requirement of *Lactobacillus* spp. in completely chemically defined growth media. *Journal of Applied Microbiology*.

[5] Wohlgemuth S, Loh G, Blaut M (2010). Recent developments and perspectives in the investigation of probiotic effects. *International Journal of Medical Microbiology*.

[6] Kühbacher T, Ott SJ, Helwig U (2006). Bacterial and fungal microbiota in relation to probiotic therapy (VSL#3) in pouchitis. *Gut*.

[7] Cleusix V, Lacroix C, Vollenweider S, Le Blay G (2008). Glycerol induces reuterin production and decreases *Escherichia coli* population in an in vitro model of colonic fermentation with immobilized human feces. *FEMS Microbiology Ecology*.

[8] Szajewska H, Kotowska M, Mrukowicz JZ, Armánska M, Mikolajczyk W (2001). Efficacy of *Lactobacillus* GG in prevention of nosocomial diarrhea in infants. *Journal of Pediatrics*.

[9] Perdigon G, Alvarez S, Rachid M, Agüero G, Gobbato N (1995). Immune system stimulation by probiotics. *Journal of dairy science*.

[10] Konstantinov SR, Smidt H, De Vos WM (2008). S layer protein A of *Lactobacillus acidophilus* NCFM regulates immature dendritic cell and T cell functions. *Proceedings of the National Academy of Sciences of the United States of America*.

[11] Arvola T, Laiho K, Torkkeli S (1999). Prophylactic *Lactobacillus* GG reduces antibiotic-associated diarrhea in children with respiratory infections: a randomized study. *Pediatrics*.

[12] Mazmanian SK, Round JL, Kasper DL (2008). A microbial symbiosis factor prevents intestinal inflammatory disease. *Nature*.

[13] Hayes KS, Bancroft AJ, Goldrick M, Portsmouth C, Roberts IS, Grencis RK (2010). Exploitation of the intestinal microflora by the parasitic nematode *Trichuris muris*. *Science*.

[14] Benson A, Pifer R, Behrendt CL, Hooper LV, Yarovinsky F (2009). Gut commensal bacteria direct a protective immune response against *Toxoplasma gondii*. *Cell Host and Microbe*.

[15] Clark DP (1999). New insights into human *Cryptosporidium*. *Clinical Microbiology Reviews*.

[16] Gargala G (2008). Drug treatment and novel drug target against *Cryptosporidium*. *Parasite*.

[17] Griffiths JK, Theodos C, Paris M, Tzipori S (1998). The gamma interferon gene knockout mouse: a highly sensitive model for evaluation of therapeutic agents against *Cryptosporidium parvum*. *Journal of Clinical Microbiology*.

[18] Mead JR, Arrowood MJ, Sidwell RW, Healey MC (1991). Chronic *Cryptosporidium parvum* infections in congenitally immunodeficient SCID and nude mice. *The Journal of Infectious Diseases*.

[19] Harp JA, Goff JP (1998). Strategies for the control of *Cryptosporidium parvum* infection in calves. *Journal of Dairy Science*.

[20] Harp JA, Chen W, Harmsen AG (1992). Resistance of severe combined immunodeficient mice to infection with *Cryptosporidium parvum*: the importance of intestinal microflora. *Infection and Immunity*.

[21] Alak JIB, Wolf BW, Mdurvwa EG, Pimentel-Smith GE, Adeyemo O (1997). Effect of *Lactobacillus reuteri* on intestinal resistance to *Cryptosporidium parvum* infection in a murine model of acquired immunodeficiency syndrome. *The Journal of Infectious Diseases*.

[22] Alak JI, Wolf BW, Mdurvwa EG (1999). Supplementation with *Lactobacillus reuteri* or *L. acidophilus* reduced intestinal shedding of *Cryptosporidium parvum* oocysts in immunodeficient C57BL/6 mice. *Cellular and Molecular Biology*.

[23] Waters WR, Harp JA, Wannemuehler MJ, Carbajal NY, Casas IA (1999). Effects of *Lactobacillus reuteri* on *Cryptosporidium parvum* infection of gnotobiotic TCR-*α*-deficient mice. *Journal of Eukaryotic Microbiology*.

[24] Pickerd N, Tuthill D (2004). Resolution of cryptosporidiosis with probiotic treatment. *Postgraduate Medical Journal*.

[25] Guitard J, Menotti J, Desveaux A (2006). Experimental study of the effects of probiotics on *Cryptosporidium parvum* infection in neonatal rats. *Parasitology Research*.

[26] Harp JA, Jardon P, Rob Atwill E (1996). Field testing of prophylactic measures against *Cryptosporidium parvum* infection in calves in a California dairy herd. *American Journal of Veterinary Research*.

[27] Glass MD, Courtney PD, LeJeune JT, Ward LA (2004). Effects of *Lactobacillus acidophilus* and *Lactobacillus reuteri* cell-free supernatants on *Cryptosporidium* viability and infectivity *in vitro*. *Food Microbiology*.

[28] Foster JC, Glass MD, Courtney PD, Ward LA (2003). Effect of *Lactobacillus* and *Bifidobacterium* on *Cryptosporidium parvum* oocyst viability. *Food Microbiology*.

[29] Deng M, Nuanualsuwan S, Cliver DO (2001). Inactivation of *Cryptosporidium parvum* oocysts by bacterial strains. *Journal of Eukaryotic Microbiology*.

[30] Ankarklev J, Jerlströ-Hultqvist J, Ringqvist E, Troell K, Svärd SG (2010). Behind the smile: cell biology and disease mechanisms of *Giardia* species. *Nature Reviews Microbiology*.

[31] Hawrelak J (2003). Giardiasis: pathophysiology and management. *Alternative Medicine Review*.

[32] Singer SM, Nash TE (2000). The role of normal flora in *Giardia lamblia* infections in mice. *The Journal of Infectious Diseases*.

[33] Perez PF, Minnaard J, Rouvet M (2001). Inhibition of *Giardia intestinalis* by extracellular factors from *Lactobacilli*: an in vitro study. *Applied and Environmental Microbiology*.

[34] Humen MA, De Antoni GL, Benyacoub J (2005). *Lactobacillus johnsonii* La1 antagonizes *Giardia intestinalis* in vivo. *Infection and Immunity*.

[35] Shukla G, Devi P, Sehgal R (2008). Effect of *Lactobacillus casei* as a probiotic on modulation of giardiasis. *Digestive Diseases and Sciences*.

[36] Benyacoub J, Pérez PF, Rochat F (2005). *Enterococcus faecium* SF68 enhances the immune response to *Giardia intestinalis* in mice. *Journal of Nutrition*.

[37] Shukla G, Sidhu RK (2011). *Lactobacillus casei* as a probiotic in malnourished *Giardia lamblia*-infected mice: a biochemical and histopathological study. *Canadian Journal of Microbiology*.

[38] Guillot CC, Bacallao EG, Dominguez MSC, Garcia MF, Gutierrez PM (1995). Effects of *Saccharomyces boullardii* in children with chronic diarrhea, especially cases due to giardiasis. *Revista Mexicana de Puericultura y Pediatria*.

[39] Besirbellioglu B, Ulcay A, Can M (2006). *Saccharomyces boulardii* and infection due to *Giardia lamblia*. *Scandinavian Journal of Infectious Diseases*.

[40] Lee SH, Lillehoj HS, Dalloul RA, Park DW, Hong YH, Lin JJ (2007). Influence of *Pediococcus*-based probiotic on coccidiosis in broiler chickens. *Poultry Science*.

[41] Shirley MW, Smith AL, Tomley FM (2005). The biology of avian *Eimeria* with an emphasis on their control by vaccination. *Advances in Parasitology*.

[42] McDonald V, Shirley MW (2009). Past and future: vaccination against *Eimeria*. *Parasitology*.

[43] Allen PC, Fetterer RH (2002). Recent advances in biology and immunobiology of *Eimeria* species and in diagnosis and control of infection with these coccidian parasites of poultry. *Clinical Microbiology Reviews*.

[44] Kabir SML (2009). The role of probiotics in the poultry industry. *International Journal of Molecular Sciences*.

[45] Dalloul RA, Lillehoj HS, Shellem TA, Doerr JA (2003). Enhanced mucosal immunity against *Eimeria acervulina* in broilers fed a *Lactobacillus*-based probiotic. *Poultry Science*.

[46] Dalloul RA, Lillehoj HS, Shellem TA, Doerr JA (2003). Intestinal immunomodulation by vitamin A deficiency and *lactobacillus*-based probiotic in *Eimeria acervulina*-infected broiler chickens. *Avian Diseases*.

[47] Dalloul RA, Lillehoj HS, Tamim NM, Shellem TA, Doerr JA (2005). Induction of local protective immunity to *Eimeria acervulina* by a *Lactobacillus*-based probiotic. *Comparative Immunology, Microbiology and Infectious Diseases*.

[48] Lee S, Lillehoj HS, Park DW, Hong YH, Lin JJ (2007). Effects of *Pediococcus*- and *Saccharomyces*-based probiotic (MitoMax) on coccidiosis in broiler chickens. *Comparative Immunology, Microbiology and Infectious Diseases*.

[49] Tierney J, Gowing H, Van Sinderen D (2004). In vitro inhibition of *Eimeria tenella* invasion by indigenous chicken *Lactobacillus* species. *Veterinary Parasitology*.

[50] Bautista-Garfias CR, Ixta-Rodríguez O, Martínez-Gómez F, Lopez MG, Aguilar-Figueroa BR (2001). Effect of viable or dead *Lactobacillus casei* organisms administered orally to mice on resistance against *Trichinella spiralis* infection. *Parasite*.

[51] Kato I, Tanaka K, Yokokura T (1999). Lactic acid bacterium potently induces the production of interleukin-12 and interferon-*γ* by mouse splenocytes. *International Journal of Immunopharmacology*.

[52] Basualdo J, Sparo M, Chiodo P, Ciarmela M, Minvielle M (2007). Oral treatment with a potential probiotic (*Enterococcus faecalis* CECT 7121) appears to reduce the parasite burden of mice infected with *Toxocara canis*. *Annals of Tropical Medicine and Parasitology*.

[53] Solano-Aguilar G, Shea-Donohue T, Madden K, Dawson H, Ledbetter T, Urban JJ, Gasbarre LC (2004). The effect of human-derived probiotic bacteria on the intestinal function of pigs. *Symposium: New Approaches in the Study of Animal Parasites*.

[54] Dawson HD, Beshah E, Nishi S (2005). Localized multigene expression patterns support an evolving Th1/Th2-like paradigm in response to infections with *Toxoplasma gondii* and *Ascaris suum*. *Infection and Immunity*.

[55] de Fátima Macedo Santos J, Vasconcelos J, de Souza JR, de Medeiros Coutinho E, Montenegro SML, Azevedo-Ximemes E (2004). The effect of *Zymomonas mobilis* culture on experimental *Schistosoma mansoni* infection. *Revista da Sociedade Brasileira de Medicina Tropical*.

[56] Bautista-Garfias CR, Gomez MB, Aguilar BR, Ixta O, Martinez F, Mosqueda J (2005). The treatment of mice with *Lactobacillus casei* induces protection against *Babesia microti* infection. *Parasitology Research*.

[57] Goff WL, Johnson WC, Tuo W (2002). Age-related innate immune response in calves to *Babesia bovis* involves IL-12 induction and IL-10 modulation. *Annals of the New York Academy of Sciences*.

[58] Bautista CR, Sandoval A, Aguilar BR (2008). Effect of high- and low-molecular-weight components of *Lactobacillus casei* on resistance against *Babesia microti* in NIH mice. *Annals of the New York Academy of Sciences*.

[59] Martinez-Gomez F, Ixta-Rodriguez O, Aguilar-Figueroa B, Hernandez-Cruz R, Monroy-Ostria A (2006). *Lactobacillus casei* ssp. rhamnosus enhances non specific protection against *Plasmodium chabaudi* AS in mice. *Salud Publica de Mexico*.

[60] Garfias CRB, Álvarez MCT, Gómez FM (2008). The inoculation of *Lactobacillus casei* in NIH mice induces a protective response against *Trypanosoma cruzi* (Ninoa strain) infection. *Veterinaria Mexico*.

[61] Maldonado Galdeano C, Perdigón G (2006). The probiotic bacterium *Lactobacillus casei* induces activation of the gut mucosal immune system through innate immunity. *Clinical and Vaccine Immunology*.

[62] Oliveira AC, Peixoto JR, de Arrada LB (2004). Expression of functional TLR4 confers proinflammatory responsiveness to *Trypanosoma cruzi* glycoinositolphospholipids and higher resistance to infection with *T. cruzi*. *Journal of Immunology*.

[63] Mansour-Ghanaei F, Dehbashi N, Yazdanparast K, Shafaghi A (2003). Efficacy of *saccharomyces boulardii* with antibiotics in acute amoebiasis. *World Journal of Gastroenterology*.

[64] Brandt LJ, Bjorkman D, Fennerty MB (2002). Systematic review on the management of irritable bowel syndrome in North America. *The American journal of gastroenterology*.

[65] Verdu EF, Collins SM (2004). Microbial-gut interactions in health and disease. Irritable bowel syndrome. *Best Practice & Research Clinical Gastroenterology*.

[66] Stark D, van Hal S, Marriott D, Ellis J, Harkness J (2007). Irritable bowel syndrome: a review on the role of intestinal protozoa and the importance of their detection and diagnosis. *International Journal for Parasitology*.

[67] Quigley EMM (2007). Bacterial flora in irritable bowel syndrome: role in pathophysiology, implications for management. *Multiphase Pumping and Technologies Conference and Exhibition 2007*.

[68] Bixquert Jiménez M (2009). Treatment of irritable bowel syndrome with probiotics. An etiopathogenic approach at last?. *Revista Espanola de Enfermedades Digestivas*.

[69] Robertson LJ, Hanevik K, Escobedo AA, Mørch K, Langeland N (2010). Giardiasis—why do the symptoms sometimes never stop?. *Trends in Parasitology*.

[70] Casas IA, Dobrogosz WJ (2000). Validation of the probiotic concept: *Lactobacillus reuteri* confers broad-spectrum protection against disease in humans and animals. *Microbial Ecology in Health and Disease*.

[71] Martin FPJ, Verdu EF, Wang Y (2006). Transgenomic metabolic interactions in a mouse disease model: interactions of *Trichinella spiralis* infection with dietary *Lactobacillus paracasei* supplementation. *Journal of Proteome Research*.

[72] O’Sullivan MA, O’Morain CA (2000). Bacterial supplementation in the irritable bowel syndrome. A randomised double-blind placebo-controlled crossover study. *Digestive and Liver Disease*.

[73] Niedzielin K, Kordecki H, Birkenfeld B (2001). A controlled, double-blind, randomized study on the efficacy of *Lactobacillus plantarum* 299V in patients with irritable bowel syndrome. *European Journal of Gastroenterology and Hepatology*.

[74] Hamilton-Miller JMT (2001). Probiotics in the management of irritable bowel syndrome: a review of clinical trials. *Microbial Ecology in Health and Disease*.

[75] Gill HS (2003). Probiotics to enhance anti-infective defences in the gastrointestinal tract. *Bailliere’s Best Practice and Research in Clinical Gastroenterology*.

[76] Lam EKY, Tai EKK, Koo MWL (2007). Enhancement of gastric mucosal integrity by *Lactobacillus rhamnosus* GG. *Life Sciences*.

[77] Borchers AT, Selmi C, Meyers FJ, Keen CL, Gershwin ME (2009). Probiotics and immunity. *Journal of Gastroenterology*.

[78] Yang Y, Iji PA, Kocher A, Thomson E, Mikkelsen LL, Choct M (2008). Effects of mannanoligosaccharide in broiler chicken diets on growth performance, energy utilisation, nutrient digestibility and intestinal microflora. *British Poultry Science*.

